# Effect of Proton Irradiation on Thin-Film YBa_2_Cu_3_O_7−δ_ Superconductor

**DOI:** 10.3390/ma17184601

**Published:** 2024-09-19

**Authors:** Joseph Fogt, Hope Weeda, Trevor Harrison, Nolan Miles, Kyuil Cho

**Affiliations:** Department of Physics, Hope College, Holland, MI 49423, USA; joseph.fogt@hope.edu (J.F.); hope.weeda@hope.edu (H.W.); nolan.miles@hope.edu (N.M.)

**Keywords:** high-temperature superconductor, cuprates, YBCO, thin film, proton irradiation, disorders, resistivity, Abrikosov–Gor’kov theory, d-wave, scattering rate

## Abstract

We investigated the effect of 0.6 MeV proton irradiation on the superconducting and normal-state properties of thin-film ${\mathrm{YBa}}_{2}{\mathrm{Cu}}_{3}O_{7-\delta }$ superconductors. A thin-film YBCO superconductor (≈567 nm thick) was subject to a series of proton irradiations with a total fluence of $7.6\times 10^{16}$ ${p/\mathrm{cm}}^{2}$. Upon irradiation, $T_{c}$ was drastically decreased from 89.3 K towards zero with a corresponding increase in the normal-state resistivity above $T_{c}$. This increase in resistivity, which indicates an increase in defects inside the thin-film sample, can be converted to the dimensionless scattering rate. We found that the relation between $T_{c}$ and the dimensionless scattering rate obtained during proton irradiation approximates the generalized d-wave Abrikosov–Gor’kov theory better than the previous results obtained from electron irradiations. This is an unexpected result, since the electron irradiation is known to be most effective to suppress superconductivity over other heavier ion irradiations such as proton irradiation. In comparison with the previous irradiation studies, we found that the result can be explained by two facts. First, the dominant defects created by 0.6 MeV protons can be point-like when the implantation depth is much longer than the sample thickness. Second, the presence of defects on all element sites is important to effectively suppress $T_{c}$.

## 1. Introduction

${\mathrm{YBa}}_{2}{\mathrm{Cu}}_{3}O_{7-\delta }$ (YBCO) is one of the most heavily studied superconductors due to its high critical temperature [1,2] and practical applications such as superconducting magnets [3] and nuclear fusion reactors [4]. While its Cooper pairing mechanism is not entirely understood, it is accepted that optimally doped YBCO ($T_{c}$ ≈ 93 K) has nodal d-wave pairing symmetry of the order parameter [5,6,7,8,9].

One useful method for determining the pairing symmetry of the order parameter is to introduce disorders into the crystal structure of a superconductor and investigate the response of its superconducting properties. Depending on the pairing symmetry, disorders will have different effects on the superconducting properties. In an isotropic s-wave superconductor, non-magnetic disorders are not effective in suppressing superconductivity (the so-called Anderson’s theorem) [10]. However, magnetic disorders are effective scatterers for suppressing the $T_{c}$ of s-wave superconductors (the Abrikosov–Gor’kov theory, or AG theory) [11]. In an anisotropic d-wave superconductor, non-magnetic disorders are also effective scatterers for suppressing superconductivity [12]. Therefore, Openov et al. developed a generalized AG theory that can include the effect of non-magnetic disorders on the property of d-wave superconductors [13].

The effect of disorders on the properties of superconductors has been experimentally investigated by conducting high-energy particle irradiations for various purposes: to enhance the critical current density $J_{c}$ [14] and to simulate space radiation environments on Earth to test various space devices, such as high-temperature superconducting microwave filters [15,16], and to study the pairing symmetry of superconductors (the purpose of the current study). Different types of high-energy particles have been used, such as electrons [17,18,19,20], protons [21,22], and heavy ions [23,24]. Electron irradiation is known to generate atomic-size point defects (due to its low rest mass) [18]; proton irradiation is known to generate a cascade (or cluster) of point defects; and heavy-ion irradiation is known to generate columnar defects. Among them, electron irradiation, which can create point-defects, is known to be the most effective in suppressing superconductivity. Indeed, the electron irradiation studies performed on YBCO compounds show qualitative agreement with the generalized d-wave AG theory. However, the results are still far from quantitative agreement. Therefore, this disagreement was explained in diverse ways, such as the quality of each sample using different plasma frequencies [12,25], the ratio between in-plane and out-of-plane defects [26], and the strong correlation [27,28,29].

To understand the relation between disorders and $T_{c}$ of d-wave superconductors, we conducted 0.6 MeV proton irradiation in a thin-film YBCO superconductor (≈567 nm thick) using Hope College’s particle accelerator (a 1.7 MV tandem Van de Graaf electrostatic accelerator). After a series of irradiation and resistance measurements, we obtained the relation between $T_{c}$ and normal-state resistivity. After converting normal-state resistivity to a dimensionless scattering rate, we compared our result with the generalized d-wave AG theory and previous electron irradiation results. It was found that our results agree better with the generalized d-wave AG theory than previous electron irradiation studies. This is an unexpected outcome, since the electron irradiation is known to be more effective in suppressing superconductivity than other forms of irradiation, such as proton irradiation. In comparison with the previous irradiation studies, we found that the current result can be explained by two facts. First, the dominant defects created by 0.6 MeV protons can be point-like when the implantation depth is much longer than the sample thickness. Second, the presence of defects on all element sites is important to effectively suppress $T_{c}$.

## 2. Materialsand Methods

### 2.1. YBCO Thin-Film Single Crystal

The YBCO thin film (≈567 nm thick) was epitaxially grown on a lanthanum aluminate (LaAlO_3_, or LAO) substrate. Photoresist was spin-coated onto the film, baked, exposed under a mask to UV light, and milled with an Ar ion beam. After patterning, the film was annealed in O_2_ at 500 °C for one hour. The sample was originally fabricated as resonators in commercial microwave filters for wireless base stations [30]. This thin-film sample shows $T_{c}$ ≈ 89.3 K, indicating that its superconducting property is close to the bulk single-crystalline sample of $T_{c}$ ≈ 93 K.

### 2.2. Resistance Measurement

**Figure 1:**
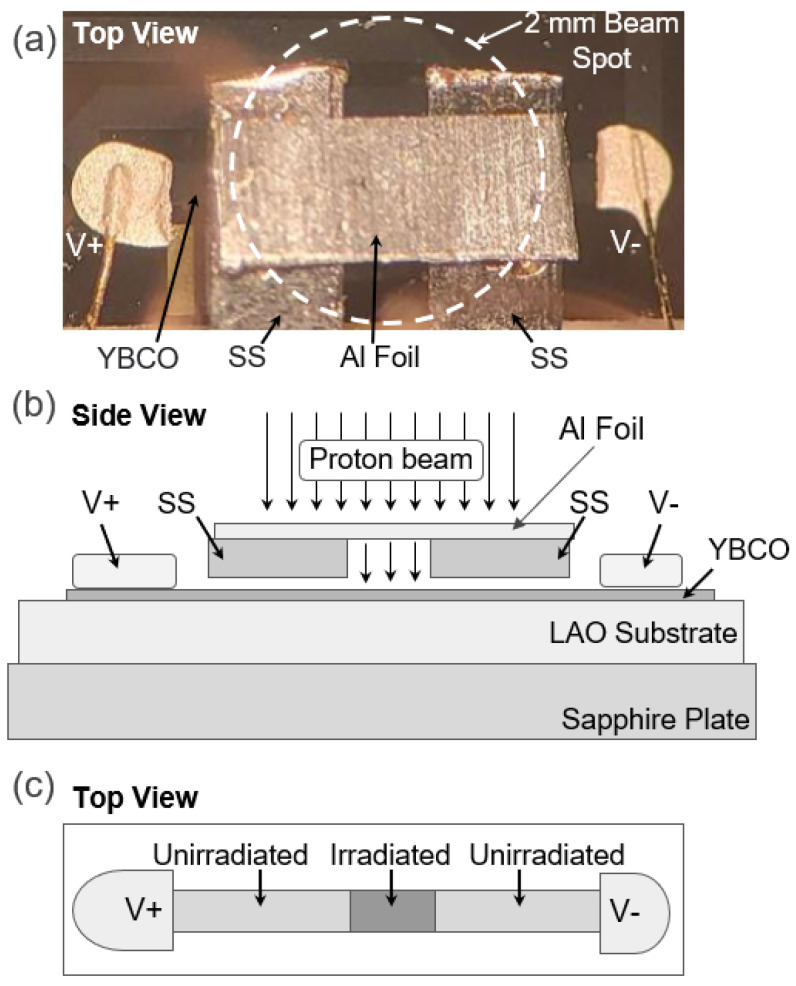
The YBCO thin-film sample patterned on LAO substrate. It is prepared for resistance measurement and proton irradiation. (**a**) The top view that shows the section where the resistance is measured between V+ and V−. I+ and I− are located outside of this image. The white dashed circle indicates a 2 mm diameter spot of the proton beam. 50 μm thick aluminum foil (energy degrader) was placed above the sample to decrease the proton beam energy from 2.2 MeV to 0.6 MeV. The degrader was placed right above two stainless-steel (SS) plates (0.19 mm thick). Since the SS plates completely block the proton beam, only the center area of the YBCO sample between the SS plates was irradiated. (**b**) A side view that shows the aluminum energy degrader and two SS blocks placed above the YBCO thin film without physical contact. The YBCO thin film was epitaxially grown on the LAO substrate. YBCO/LAO was attached to a sapphire plate using silver paste. (**c**) A schematic diagram that shows the areas of irradiated and unirradiated parts of the YBCO thin film. Proton irradiation was applied only to the center part of the thin film.

**Figure 2:**
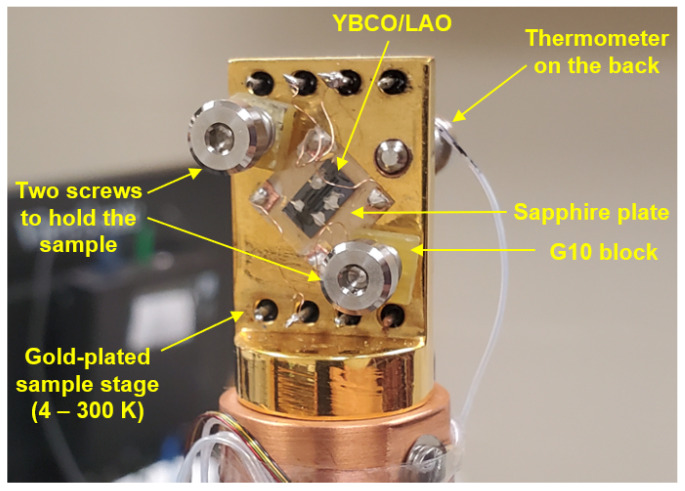
YBCO/LAO sample mounted on a 4K cryogen-free cryostat (CS-202SE, Advanced Research Systems, Inc., Macungie, PA, USA). Two screws hold the sapphire plate to which the YBCO/LAO sample is attached using silver paste.

The in-plane resistance of the YBCO thin film was measured using a standard four-probe technique. Figure 1a shows the part of the sample where the resistance is measured between V+ and V−. I+ and I− are located outside of the image. The dimensions of the measured part of the sample are 2.663 (±0.016) mm × 0.2570 (±0.0008) mm × 566.7 (±1.9) nm. Four electrical contacts made of thin gold wires were adhered to the thin film using silver paste. The YBCO/LAO sample was attached to a sapphire plate using silver paste, and the whole setup was mounted on the gold-plated sample stage of a 4K cryostat with two screws (Figure 2) for temperature-dependent resistance measurements.

### 2.3. Energy Degrader

The Hope College’s particle accelerator can directly generate a proton beam of energy ranging from 0.6 MeV to 3.4 MeV. However, the low-energy mode at 0.6 MeV is unsafe for a long period of operation since the low-energy beam can unintentionally damage the beam line. Therefore, we developed an alternative way to generate a 0.6 MeV proton beam. We first generated a 2.2 MeV proton beam and then passed this beam through an aluminum energy degrader to decrease the beam energy from 2.2 MeV to 0.6 MeV.

To accomplish this, we conducted a series of proton irradiations directly onto an aluminum degrader (50 μm foil, measured by a micrometer) by varying the energy from 1.9 to 2.6 MeV, and measured the beam current after the beam passed through the aluminum degrader using a Faraday cup. It was found that proton beams of energies lower than 2.0 MeV weren’t able to pass through the degrader. This suggests that the penetration distance of the proton beam is smaller than the thickness of the aluminum degrader when the accelerator energy is lower than 2.0 MeV. Using SRIM software (http://www.srim.org/ accessed on 1 May 2024) [31], it is found that the effective thickness of the aluminum degrader corresponding to 2.0 MeV is 41.63 μm (smaller than 50 μm, measured by the micrometer).

**Figure 3:**
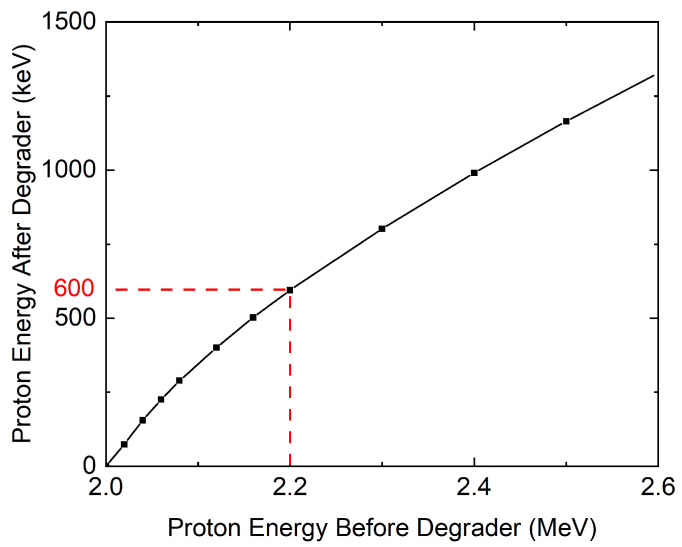
Proton energy before and after an aluminum energy degrader, calculated using SRIM software (http://www.srim.org/ accessed on 1 May 2024). The red dotted line indicates the energy used for the current study (about 600 keV).

The effective thickness of the degrader (41.63 μm) was used to calculate the beam energies after degradation by the aluminum degrader, as shown in Figure 3. For example, the projected penetration distance for a 2.2 MeV proton into an infinitely thick aluminum degrader is 48.49 μm according to SRIM. Since the effective thickness of the aluminum degrader is 41.63 μm, the beam still has some energy left after traveling through the target. Thus, the difference between 48.49 μm and 41.63 μm (i.e., 6.86 μm) is the distance that a proton may travel after interacting with the aluminum target. This value can be converted to the energy after degradation, which is 0.6 MeV in the case of a 2.2 MeV beam. This process was repeated for multiple beam energies and the resulting data were plotted in Figure 3.

### 2.4. Homogeneous Proton Beam

**Figure 4:**
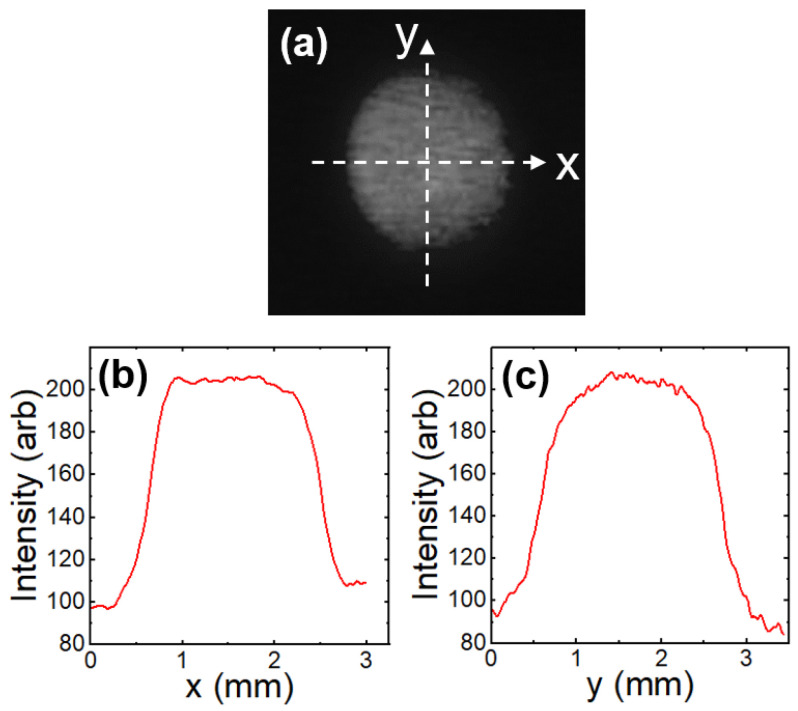
(**a**) The 2 mm diameter proton beam projected on a Mylar scintillator. (**b**,**c**): Intensity of the beam along the *x* and *y* axes.

The 0.6 MeV proton irradiation was conducted at room temperature in a vacuum chamber of $6\times 10^{-8}$ torr. The beam was directed along the *c*-axis of the YBCO thin-film sample. We used a 2 mm diameter aperture to generate a 2 mm diameter proton beam. The beam profile shown in Figure 4 indicates that its center area is homogeneous. The actual positions of YBCO sample, the 2 mm beam spot, and the aluminum energy degrader are described in Figure 1a. In addition, two 0.19 mm-thick stainless-steel (SS) plates were placed above the sample without physical contact. Since the proton beam cannot penetrate the SS plates, the irradiation only affected the area between two SS plates. The irradiated part of the YBCO thin film (0.591 (±0.013) mm × 0.257 (±0.0008) mm × 566.7 (±1.9) nm), which is much smaller than the 2 mm diameter beam spot, was positioned at the center of the beam to experience the most homogeneous beam (Figure 1). Figure 1c is a schematic diagram that shows where the irradiation was performed. In this way, we generated a homogeneous 0.6 MeV proton beam on the well-defined section of the YBCO thin film. The current of the beam was measured using a Faraday cup every 30 min during the irradiation. The current of the 2 mm diameter beam remained stable at 10 nA (±2 nA) during the whole irradiation process. To avoid sample heating, the current was kept below 12 nA. After irradiation, the sample was transferred from the accelerator vacuum chamber to the cryostat (Figure 2) for resistance measurement.

## 3. Results and Discussion

**Figure 5:**
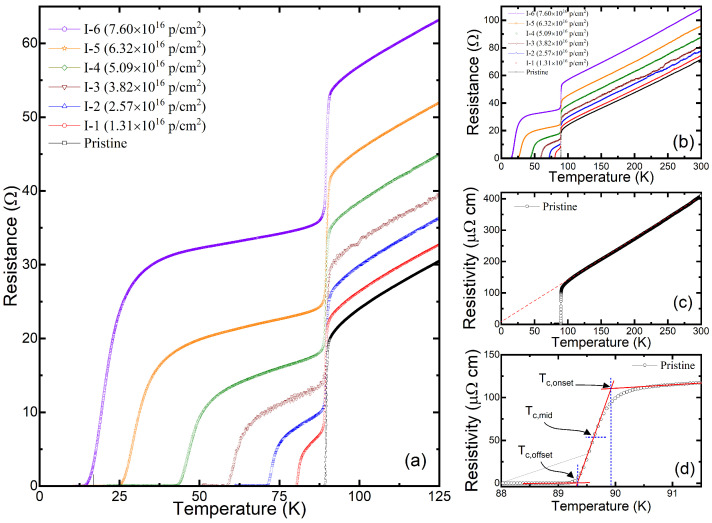
(**a**) The temperature-dependent resistance measured in the YBCO thin-film sample upon a series of irradiation processes (from I-1 to I-6). The transition regions between 0 K and 125 K are shown. The data in (**b**) are identical to the panel (**a**), which includes the data up to 300 K. (**c**) The temperature-dependent resistivity of the pristine sample. (**d**) A magnification of the panel (**c**) near the superconducting transition. Among the different definitions of $T_{c}$, we used the offset definition ($T_{c,offset}$) throughout this article.

**Figure 6:**
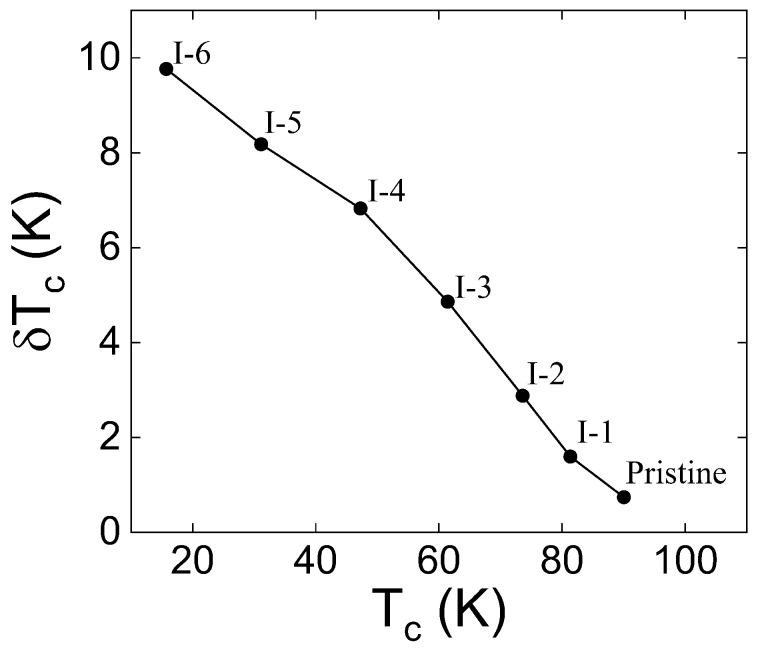
$\delta T_{c}$ = $T_{c,onset}-T_{c,offset}$ that shows the broadening of the superconducting transition. It increases upon proton irradiation.

We performed seven resistance measurements alternated with six proton irradiations in the identical YBCO sample. Figure 5a,b show all the resistance measurements. Except for the pristine case, all the other data show two superconducting transitions. Two superconducting transitions are observed, since the irradiation was only performed on the center part of the YBCO sample, as shown in Figure 1c. Therefore, the $T_{c}$ of the irradiated part decreases upon irradiation while the $T_{c}$ of the unirradiated part remains unchanged. Two-step transitions are not seen for the pristine case because its entire area has not been irradiated. In the irradiated portion of the sample, the damage to superconducting properties is evident, with the $T_{c}$ decreasing rapidly from the initial $T_{c}$ of 89.3 K towards zero. Furthermore, the transitions get broader as the fluence increases as shown in Figure 6. Figure 5c is the temperature-dependent resistivity of the pristine sample calculated based on the shape of the sample. The linear approximation of the normal-state resistivity suggests a very small residual resistivity (≈8 μΩcm) at *T* = 0 K. Figure 5d shows $T_{c,offset}$, which is used for data analysis.

Figure 5a,b also show that upon proton irradiation, the normal-state resistance above $T_{c}$ monotonically increases over the entire high-temperature region. This parallel upward shift in resistance is consistent with Matthiessen’s rule, suggesting that the number of defects in the YBCO sample gradually increases upon irradiation. In addition, this increase in resistance originates from the irradiated part of the sample, while the resistance in the unirradiated part remains unchanged. Using the exact volume of the irradiated part of the sample, the resistance increase can be converted to the resistivity increase (Δρ) of the irradiated part. Since the normal-state resistance increase is almost identical in all temperature regions above $T_{c}$, the resistance value at 125 K was used to represent the increase in defects and to calculate the resistivity increase at 125 K ($\Delta \rho_{125K}$).

The generalized AG theory formulated by Openov [25] can be written for the case of the non-magnetic disorders in d-wave superconductors as follows:

(1) $$-ln\left(t_{c}\right)=\Psi \left(\frac{1}{2}+\frac{g}{2\;t_{c}}\right)-\Psi \left(\frac{1}{2}\right),$$

where $t_{c}$ is $T_{c}$/$T_{c0}$, $T_{c0}$ is the initial $T_{c}$ before the disorders are added, and *g* is the dimensionless scattering rate. $t_{c}$ asymptotically goes to zero as *g* approaches 0.28. Using the Drude model [32,33], *g* can also be written in terms of the residual resistivity ($\rho_{0}$) as follows:

(2) $$g=\frac{\hbar \;\rho_{0}}{2\pi k_{B}\mu_{0}T_{c0}\lambda_{0}^{2}},$$

where $\rho_{0}$ is the residual resistivity at *T* = 0 K of the irradiated part of the YBCO sample, $T_{c0}$ is the critical temperature of the pristine sample, and $\lambda_{0}$ is the zero-temperature London penetration depth of the pristine sample. Due to the high $T_{c}$ of the YBCO sample, it is difficult to estimate the exact residual resistivity. However, the temperature-dependent resistivity in Figure 5c shows that the linear approximation of normal-state resistivity suggests very a small residual resistivity at *T* = 0 K (≈8 μΩcm). Assuming that the $\rho_{0}$ of the pristine sample is very small, $\rho_{0}$ in Equation (2) is replaced with Δρ, and *g* can be rewritten as follows:

(3) $$g\approx \frac{\hbar \;\Delta \rho_{125K}}{2\pi k_{B}\mu_{0}T_{c0}\lambda_{0}^{2}},$$

where $\Delta \rho_{125K}$ is the resistivity increase at *T* = 125 K of the irradiated part of the YBCO sample. Since $\lambda_{0}$ varies in different studies, such as 1460±150 Å [34], 1550 Å [35], and 1990±200 Å [36], we used the most commonly accepted value of 1405±92 Å [37].

**Figure 7:**
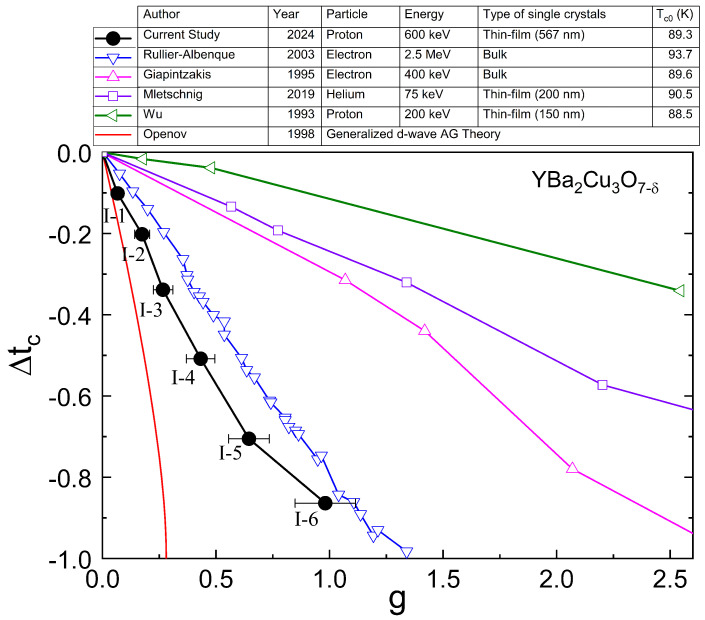
$\Delta t_{c}$ = ($T_{c}-T_{c0}$)/$T_{c0}$ as a function of dimensionless scattering rate (*g*) upon irradiation. The current proton irradiation result is compared with previous results [17,18,21,38] and the generalized d-wave AG theory [25].

Figure 7 summarizes the relation between $T_{c}$ and *g* of the current study in comparison with previous irradiation studies [17,18,21,38] and theoretical expectation [25]. The current proton irradiation study shows that $T_{c}$ linearly decreases down to $\Delta t_{c}$ ≈ −0.7 (I-5) with increasing *g*. After I-5 irradiation, however, the decrease rate slows down for I-6. Comparing the current study with previous irradiation studies on YBCO single-crystals (bulk sample and thin-film), it is evident that the current study of proton irradiation on YBCO thin film is closest to the theoretical expectation (generalized d-wave AG theory by Openov [25]). It is interesting to find that the current results show a faster suppression rate of $T_{c}$ than that of the electron irradiation study performed on a bulk single-crystalline YBCO by Rullier-Albenque et al. [17]. This outcome is unexpected, since the electron irradiation has been commonly known to be most effective in suppressing $T_{c}$ by producing atomic-size point-like defects, while other heavier ion irradiations produce less effective cluster or columnar defects.

To understand this unexpected outcome, we first compare two previous electron irradiation studies shown in Figure 7. The 2.5 MeV electron irradiation study [17] shows a much faster $T_{c}$ suppression rate than the 400 keV electron irradiation study [18]. The 400 keV electrons can create only oxygen-site point-defects due to their low energy, while 2.5 MeV electrons can create point-defects on all element sites (Y, Ba, Cu, and O). This difference can be understood by considering the energy-dependent scattering cross-sections (refer to the graph in the reference [20]). For 400 keV electrons, the partial cross-section of oxygen has a finite value, while the partial cross-sections of other elements (Y, Ba, and Cu) are almost zero. For 2.5 MeV electrons, however, all partial cross-sections have finite values. Therefore, this comparison clearly shows that the presence of defects on all element sites are important to effectively suppress $T_{c}$. Surprisingly, the current proton irradiation study shows even faster $T_{c}$ suppression than the 2.5 MeV electron irradiation study. Following the two electron irradiation comparison above, this can be now understood by the fact that 600 keV protons used in the current study can create defects on all element sites much more effectively than high-energy electrons, since the rest mass of the proton is much higher than that of the electron.

Another interesting insight comes from the consideration of the ratio between the implantation depth and the sample thickness. In general, protons are known to create cluster defects. However, the defects created by protons can also be dominated by point-like defects when the energy of protons is high enough, so the implantation depth is much larger than the sample thickness. In this case, the protons cannot create cluster defects on the sample, since cluster defects are mainly created around the implantation depth but the sample is much thinner than the implantation depth. Therefore, point-like defects dominate in this case. As shown in Figure 7, indeed, the current 600 keV proton irradiation study (567 nm thin-film YBCO) shows a much faster suppression of $T_{c}$ than the 200 keV proton irradiation study (150 nm thin-film YBCO) by Wu et al. [21]. This is due to the fact that the implantation depth of 600 keV protons (≈3.9 μm) is 6.9 times longer than the thickness of YBCO sample used in the current study. Therefore, these protons are prone to creating point-like defects without producing many cluster defects. According to the low-energy proton irradiation study by Huang et al. (60 keV protons on 500 nm YBCO thin film), however, the low-energy protons stopped in the interior of YBCO thin film due to the short implantation depth (≈349 nm) and created a large amount of 10 nm size cluster defects as well as 2 nm point defects [14]. They also claimed dominance of the oxygen site defects. Another study of 200 keV proton irradiation on 150 nm YBCO thin film also shows the dominance of cluster defects due to the low energy protons and the slow suppression of $T_{c}$ [21]. However, these low-energy proton irraditions mainly create cluster defects that are inhomogeneous along the thickness. To avoid inhomogeneity and cluster defects, we used higher energy protons (0.6 MeV), since the purpose of the current study is to find the relation between disorder and $T_{c}$ of d-wave superconductor. The 75 keV helium irradiation study on 200 nm YBCO thin film also shows a much slower $T_{c}$ suppression due to the dominance of cluster defects and short implantation depth [38].

A noticeable departure from this trend occurs for I-6 irradiation. $T_{c}$ decreases at a slower rate for I-6 than the others (from I-1 to I-5). This indicates a development of extended defects, where the nearby point defects agglomerate at high fluence, as mentioned by Wu et al. [21]. In addition, we noticed a height increase in the area of the irradiated part of the YBCO thin film by about 15 nm (identified using Atomic Force Microscopy). This increase was caused by the implantation of protons into the LAO substrate (the implantation depth of protons into YBCO/LAO = 3.9 μm from TRIM simulation [31]). A height increase upon irradiation was also observed by Zhao et al. [39]. The height increase can cause the distortion of the YBCO thin film by increasing the extra resistivity at the boundary between the irradiated part and unirradiated part of YBCO thin film. Further investigations are needed to check the influence of height increase on the suppression rate of $T_{c}$.

**Figure 8:**
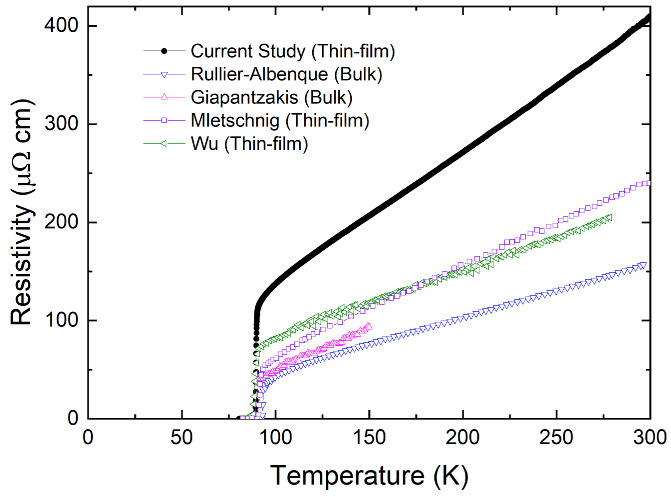
Comparison of temperature-dependent resistivities measured in different YBCO single crystals [17,18,21,38]. In general, the resistivities of thin-film samples are larger than those of bulk single-crystalline samples.

Figure 8 compares the temperature-dependent resistivities of various YBCO samples before particle irradiations. Thin-film single-crystalline samples show higher resistivity than bulk single-crystalline samples. In particular, the sample of the current study shows higher resistivity and lower $T_{c}$ than those of the previous studies, suggesting that thin films may already have strong pinning defects before irradiations. Further investigations are needed to study the effect of proton irradiation by varying the initial conditions of the sample, such as different $T_{c0}$, resistivity, and thickness.

## 4. Conclusions

We studied the effect of 0.6 MeV proton irradiation in a YBCO thin-film superconductor and found that the relation between $T_{c}$ and the dimensionless scattering rate *g* obtained from the current study approximated the generalized d-wave Abrikosov–Gor’kov theory, surpassing the previous electron irradiation studies. In comparison with the previous irradiation studies, we found that this unexpected result can be explained by two facts. First, the dominant defects created by 0.6 MeV protons can be point-like when the implantation depth is much longer than the sample thickness. Second, the presence of defects on all element sites is important to effectively suppress $T_{c}$. Further investigations with different energy proton irradiation studies and different thickness samples are needed to fully understand the relation between disorders and superconducting properties.

## Data Availability

Data are contained within the article.
